# Two-Point Versus Three-Point Fixation in Zygomaticomaxillary Complex (ZMC) Fractures: A Narrative Review

**DOI:** 10.7759/cureus.85659

**Published:** 2025-06-09

**Authors:** Rishabh Kasrija, Himanshi Gupta, Tarun Mittal, Mansi Dang, Riya Behl, Munish Kumar, Seema Gupta

**Affiliations:** 1 Department of Oral and Maxillofacial Surgery, Jagadguru Sri Shivarathreeshwara Dental College, Mysuru, IND; 2 Department of Oral and Maxillofacial Surgery, Guru Nanak Dev Dental College and Research Institute, Sunam, IND; 3 Department of Orthodontics, Kothiwal Dental College and Research Centre, Moradabad, IND

**Keywords:** fixation, fractures, review, technique, zygomaticomaxillary

## Abstract

Zygomaticomaxillary complex (ZMC) fractures are among the most prevalent facial injuries encountered during maxillofacial surgery because of the anatomical prominence and structural significance of the zygoma. These fractures often result from high-energy impacts, such as road traffic accidents, assaults, falls, and sports injuries, predominantly affecting males in their third to fourth decades of life. The management of ZMC fractures requires restoring facial aesthetics, orbital integrity, and masticatory function while minimizing surgical morbidity. Open reduction and internal fixation (ORIF) remains the primary approach, with two- and three-point fixation being the most commonly employed techniques.

This narrative review evaluates and compares the clinical outcomes, stability, aesthetic results, and complications associated with two- and three-point fixation methods. Three-point fixation, involving stabilization at the frontozygomatic suture, infraorbital rim, and zygomaticomaxillary buttress, offers superior mechanical stability, particularly in displaced or comminuted fractures. It provides better control over rotational displacement and ensures improved malar projection and facial symmetry. However, this approach is invasive and may lead to increased surgical time and tissue trauma. Two-point fixation, typically involving the frontozygomatic suture combined with either the infraorbital rim or zygomaticomaxillary buttress, is less invasive and is suitable for minimally displaced or non-comminuted fractures. It is associated with a reduced operative time and morbidity, although it may offer less control over rotational forces and result in a higher incidence of malar asymmetry in complex fractures. Although both techniques showed comparable complication rates in select cases, three-point fixation demonstrated better outcomes in terms of malar height restoration and stability under functional loads. Alternative fixation strategies, such as single- or four-point fixation, are reserved for specific clinical scenarios but carry their own limitations and risks. The decision-making process must consider the fracture classification, displacement severity, and patient-specific factors. Ultimately, individualized treatment planning, guided by clinical assessment and evidence-based practice, is essential.

Three-point fixation remains the standard for complex fractures, whereas two-point fixation is a reliable option for simpler injuries, offering a balance between functional recovery and minimal invasiveness. This review aims to evaluate and compare the clinical outcomes, stability, aesthetic results, and complications of two-point versus three-point fixation techniques in the management of zygomaticomaxillary complex fractures, guiding evidence-based treatment decisions.

## Introduction and background

Zygomaticomaxillary complex (ZMC) fractures are among the most prevalent (13%-40%) facial injuries encountered in maxillofacial surgery, primarily due to the zygoma’s prominent midfacial position [1]. The zygoma, a quadrangular bone, forms critical articulations with the maxilla, frontal, temporal, and sphenoid bones, serving as a cornerstone for facial aesthetics, orbital integrity, and masticatory function [1].

They predominantly affect young adult males (male-to-female ratio 3:1 to 5:1), with peak incidence in the 20-40 age group, driven by high-risk activities. Road traffic accidents (30-50%) and assaults (20-40%) are the leading causes, followed by falls and sports injuries. A 2017-2018 study from India reported ZMC fractures in 27.2% of 1,192 trauma cases [2]. Its exposed location makes it highly susceptible to high-energy trauma from road traffic accidents, assaults, sports injuries, and falls, with young adult males in their third to fourth decades being the most affected demographic [3]. ZMC fractures frequently result in aesthetic deformities, such as malar flattening and facial asymmetry, as well as functional impairments, including trismus, infraorbital nerve paresthesia, and diplopia, all of which significantly impact a patient’s quality of life [3]. If not properly managed, these fractures can disrupt orbital volume, leading to complications like enophthalmos or orbital dystopia, which further compromise both form and function [4].

The primary goals of ZMC fracture treatment are to achieve precise anatomical reduction, restore midfacial symmetry, preserve orbital volume, and re-establish functional occlusion [4]. Open reduction and internal fixation (ORIF), utilizing titanium plates and screws, is the cornerstone of surgical management, tailored to the fracture’s complexity and displacement severity [4]. Among the various fixation techniques, three-point fixation, which stabilizes the frontozygomatic suture, infraorbital rim, and zygomaticomaxillary buttress, is widely regarded as the gold standard for complex or displaced fractures due to its superior mechanical stability [5].

This approach ensures robust control over rotational forces and enhances malar projection, but it is more invasive, potentially increasing operative time and tissue trauma. Conversely, two-point fixation, typically involving the frontozygomatic suture paired with either the infraorbital rim or zygomaticomaxillary buttress, offers a less invasive alternative for non-comminuted or minimally displaced fractures [5]. This technique reduces surgical morbidity and operative duration but may provide less stability in complex cases, risking malar asymmetry.

This narrative review synthesizes evidence from recent literature to compare the clinical outcomes, stability, aesthetic results, and complications of two-point versus three-point fixation in ZMC fracture management, aiming to guide evidence-based surgical decision-making for optimal patient outcomes.

## Review

Methodology

A comprehensive literature search was conducted to identify studies published between January 2000 and December 2024 to ensure a broad and current evidence base. The search encompassed multiple electronic databases, including PubMed, Web of Science, peer-reviewed medical literature, Scopus for interdisciplinary surgical studies, Google Scholar for grey literature and open-access articles, Cochrane Library for systematic reviews and meta-analyses, and institutional libraries for specialized journals.

Keywords and Medical Subject Headings (MeSH) terms were strategically combined to maximize coverage. Keywords included “Zygomaticomaxillary complex,” “ZMC fractures,” “zygomatic fracture,” “two-point fixation,” “three-point fixation,” “malar asymmetry,” “facial fractures,” “open reduction internal fixation,” “orbital volume,” “infraorbital nerve,” and “aesthetic outcomes.” MeSH terms comprised “Zygomatic Fractures,” “Facial Bones/surgery,” “Fracture Fixation, Internal,” “Maxillofacial Injuries,” “Orbital Fractures,” and “Surgical Procedures, Operative.” Boolean operators (AND, OR, NOT) were used to refine the search, with a sample PubMed query structured as: (“Zygomatic Fractures” [MeSH] OR “Zygomaticomaxillary complex” OR “ZMC fractures”) AND (“Fracture Fixation, Internal” [MeSH] OR “two-point fixation” OR “three-point fixation”) AND (“Maxillofacial Injuries” [MeSH] OR “malar asymmetry” OR “aesthetic outcomes”). The filters restricted the results to English-language publications, human studies, and the specified timeframe. Manual searches of reference lists from key articles supplemented the electronic search.

This narrative review included studies published in English between January 1, 2000, and December 31, 2024, encompassing clinical trials, observational studies, systematic reviews, meta-analyses, and case series with five or more patients that either directly compared two-point versus three-point fixation or evaluated the outcomes of either technique in adult patients (aged ≥18 years) with isolated ZMC fractures requiring surgical intervention. Isolated ZMC fractures were defined as fractures involving the zygomaticomaxillary suture, zygomaticofrontal suture, or zygomatic arch, without significant concomitant facial or craniofacial fractures requiring separate surgical management. Studies were required to report at least one of the following outcomes: malar symmetry (assessed clinically or radiographically), fracture stability (e.g., absence of post-operative displacement), complications (e.g., infection, hardware failure, nerve injury), operative time, or aesthetic/functional results (e.g., facial contour, ocular function). Studies were excluded if they were published in languages other than English, involved pediatric patients (aged < 18 years), included patients with syndromic craniofacial deformities, focused on non-surgical management of ZMC fractures, were case reports or case series with fewer than five patients, or lacked specific data on ZMC fracture outcomes. The time frame of January 1, 2000, to December 31, 2024, was selected for this narrative review to include studies reflecting modern surgical techniques, materials, and imaging advancements (e.g., titanium plates, CT-guided surgery) relevant to current practice. Starting from the year 2000 excludes outdated methods, while the 2024 cutoff captures the latest evidence, ensuring relevance and applicability over a practical 25-year period. The goal of incorporating retrospective studies was to complement prospective data by providing insights into long-term outcomes, clinical practices across diverse settings, and potential complications in larger patient cohorts, thereby enhancing the review’s applicability to clinical decision-making and identifying areas for future research.

**Figure 1:**
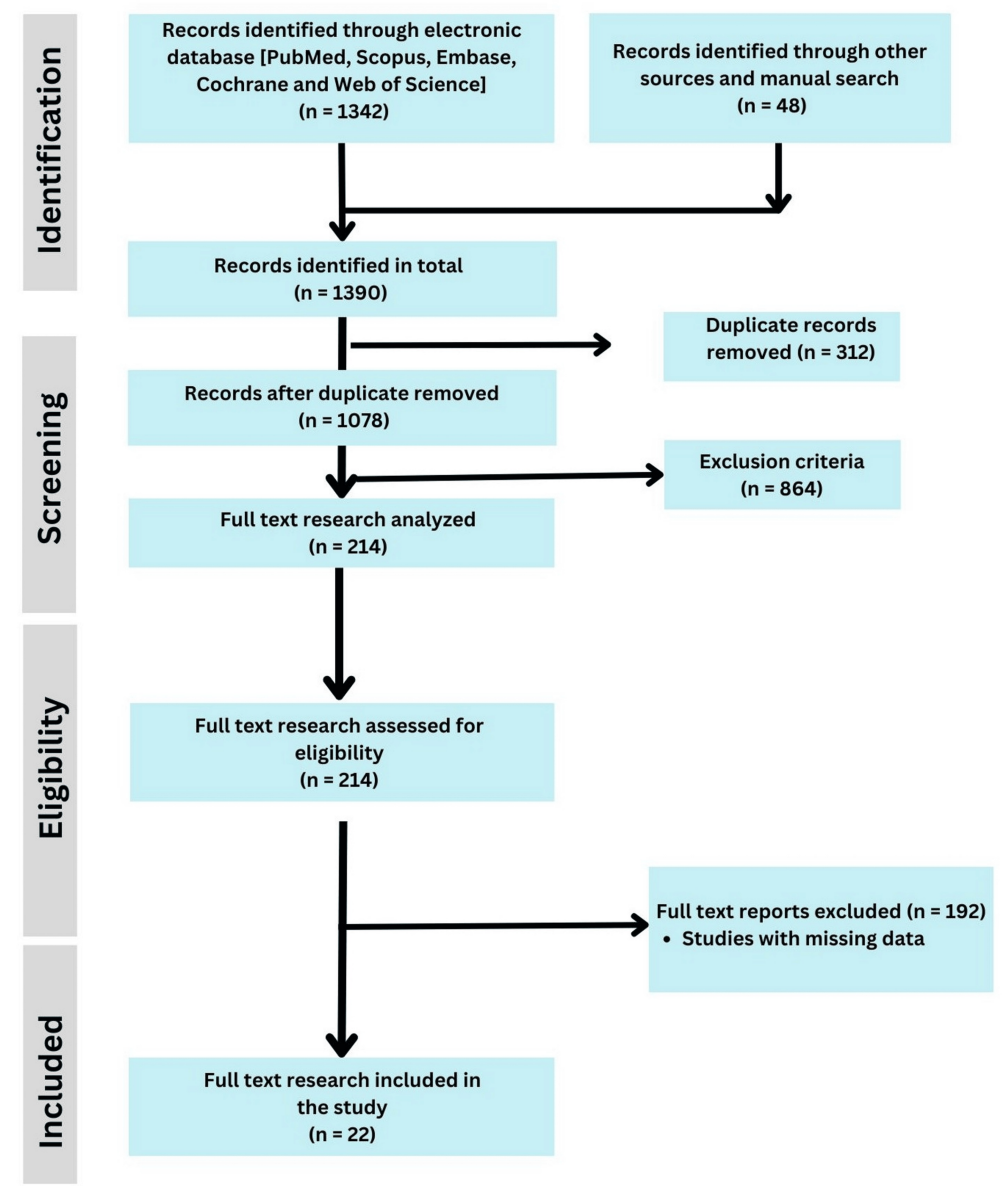
PRISMA flowchart

The selection process followed an adapted Preferred Reporting Items for Systematic Reviews and Meta-Analyses (PRISMA) framework, with initial searches yielding 1,342 articles, supplemented by 48 articles from manual searches. After removing 312 duplicates, 1,078 articles were screened by title and abstract, 214 underwent full-text review, and 22 were included (1 randomized trial, 6 prospective cohorts, 13 retrospective studies, and 2 systematic reviews). Data on study characteristics, patient demographics, fracture details, fixation techniques, and outcomes were extracted (Figure 1).

Classification of ZMC fractures

Accurate classification of ZMC fractures is essential to determine an appropriate fixation strategy. Rowe and Williams’ classification categorizes fractures based on their rotational axis and stability post-reduction [6]. Stable fractures included those limited to the zygomatic arch with medial displacement or those with medial/lateral rotation about the vertical axis. Unstable fractures encompass arch fractures with inferior displacement, ZMC fractures rotated on the horizontal axis, en bloc dislocations, and comminuted fractures. 

An alternative classification based on trauma energy divides fractures into low-energy (undisplaced or minimally displaced), medium-energy (displaced with or without fragmentation), and high-energy (massively displaced or fragmented) types [7]. Zingg et al.’s 1992 classification further delineates fractures into Type A (partial fractures involving one articulation, e.g., zygomatic arch, lateral orbital wall, or infraorbital rim), Type B (tetrapod fractures involving all four articulations), and Type C (multi-fragment fractures with zygoma body involvement).

The Knight and North (1961) [9] classification categorized ZMC fractures into six groups based on displacement and rotation. Group 1 included patients with undisplaced fractures with no significant deviation. Group 2 included isolated displaced fractures with a single shifted segment. Group 3 included displaced and unrotated body fractures. Group 4 comprised medially rotated fractures, split into Group 4a (outward displacement at the malar buttress) and Group 4b (inward displacement at the frontozygomatic suture). Group 5 included laterally rotated fractures divided into Group 5a (upward at the infraorbital margin) and Group 5b (outward at the frontozygomatic suture). Group 6 included fractures with additional lines across the main fragment, indicating greater complexity. This system helps guide surgical planning by defining the fracture characteristics. These systems inform surgical planning by highlighting the fracture complexity and stability requirements.

Patient demographics and etiology

Patients with ZMC fractures typically fall within a demographic group prone to high-impact trauma. The mean age across studies ranged from 25 to 42 years, with most patients in their third or fourth decade, reflecting exposure to activities associated with road traffic accidents and physical assault [10]. Men are predominantly affected, likely due to higher engagement in high-risk behaviors [10]. The primary etiologies include road traffic accidents, assaults, falls, and sports injuries, with regional variations in prevalence [3,11]. These demographic and etiological patterns underscore the need for tailored surgical approaches that account for patient-specific factors and injury mechanisms.

Comparison of fixation techniques

**Figure 2:**
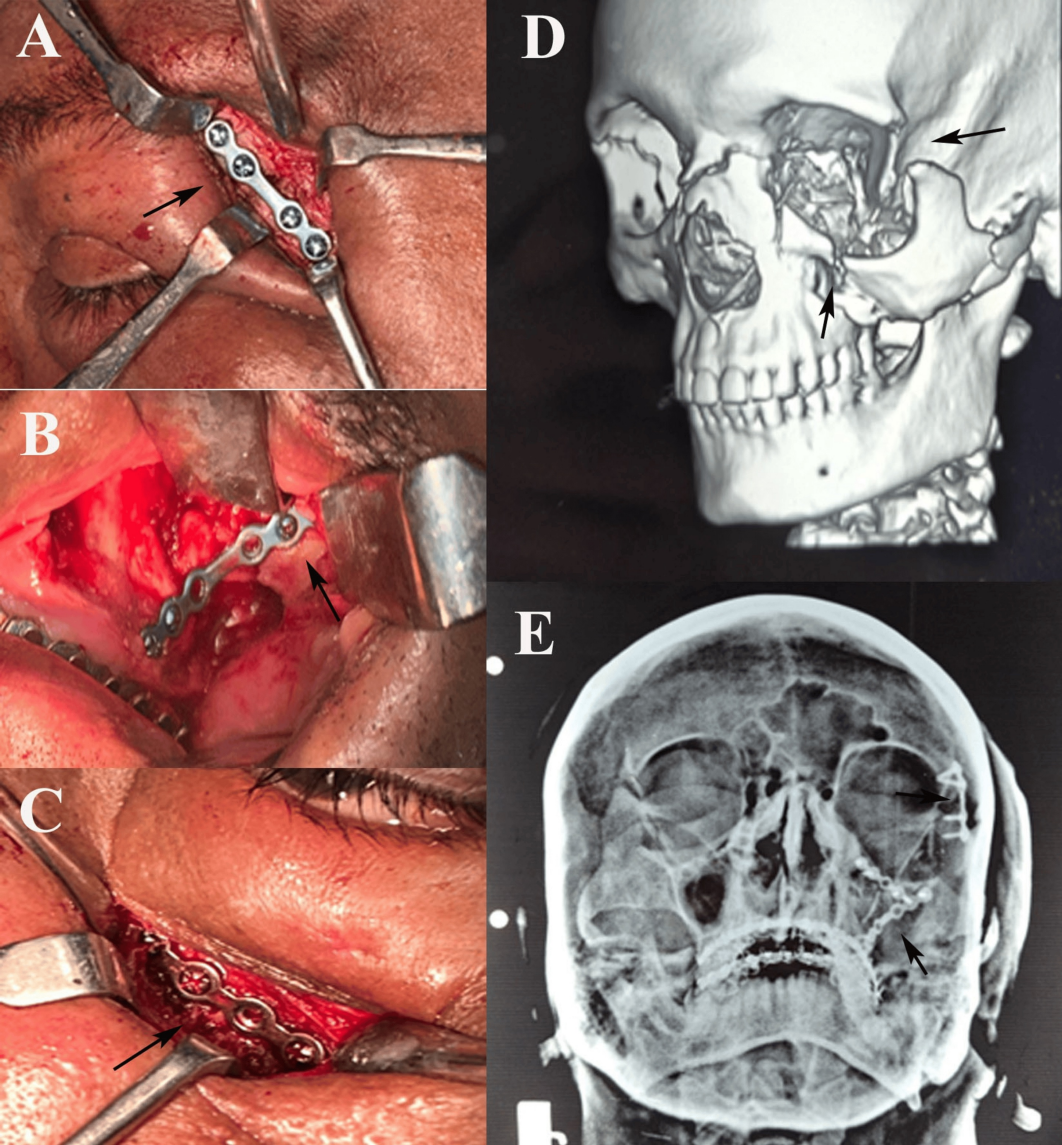
Three-point fixation using 1.5 mm titanium miniplates on (A) frontozygomatic region, (B) zygomaticomaxillary region, (C) infraorbital region, (D) preoperative CT (3D reconstruction), (E) postoperative Waters' view. All images are original and provided with consent from the patient.

The debate over two-point versus three-point fixation centers on balancing stability with surgical invasiveness [5]. Three-point fixation stabilizes the zygoma at the frontozygomatic suture, infraorbital rim, and zygomaticomaxillary buttress, addressing multiple force vectors to resist rotation, vertical displacement, and medial collapse [12]. Biomechanical studies confirm its superior resistance to masticatory and functional loads, particularly in comminuted or displaced fractures [5]. A systematic review by Gadkari et al. [12] found that three-point fixation enhances stability and malar projection in complex fractures. Jazayeri et al.’s [13] meta-analysis reported significantly lower postoperative displacement with three-point fixation than with two-point fixation. A randomized trial by Rana et al. [14] demonstrated better facial symmetry and zygomatic projection with three-point fixation, whereas Gawande et al. [15] noted improved malar eminence restoration and reduced rotation. Nasr et al. [16] further supported three-point fixation for complex fractures, citing better aesthetic and functional outcomes (Figure 2).

**Figure 3:**
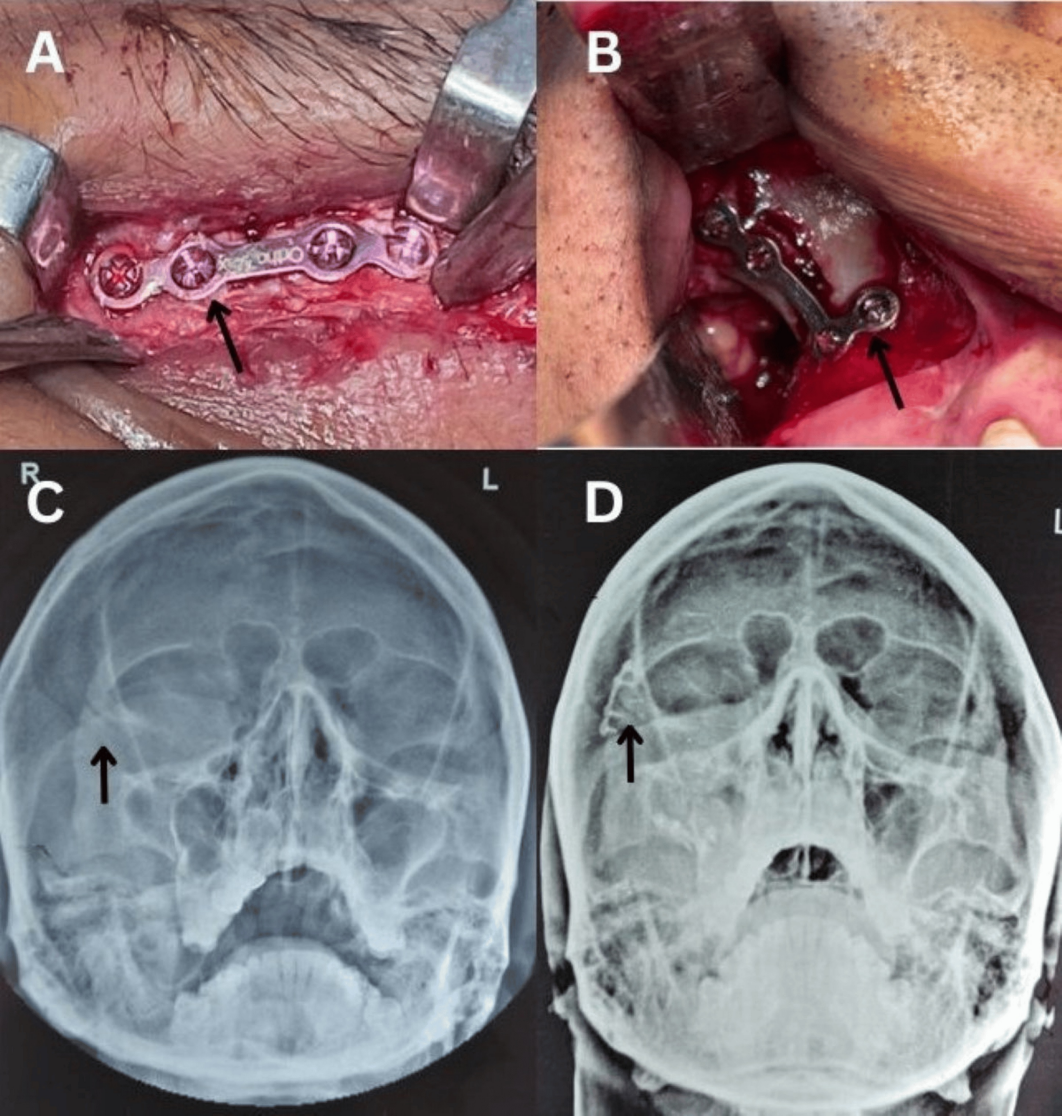
Two-point fixation using 1.5 mm titanium miniplates on (A) Frontozygomatic region, (B) Zygomaticomaxillary region, (C) Preoperative Water’s view, (D) Postoperative Water’s view. All images are original and provided with consent to the patient.

Kim et al. [17] conducted an evaluation involving 40 patients, subsequently categorizing them into two distinct groups that received two-point and three-point fixations, respectively, which were assessed over a duration of 12 weeks. The study concluded that there was negligible variance in post-operative stability between the two cohorts; therefore, the extent of displacement should not be regarded as a critical factor when determining the fixation technique, which encompasses the quantity and placement of mini-plates used for fixation. A retrospective analysis performed by Ji et al. [18] involving 502 patients over a span of 10 years determined that minimal incision techniques, along with a familiar approach and the surgeon’s preferred fixation method, are advocated for the management of ZMC fractures. In 2002, Parashar et al. [19] conducted a research investigation involving 22 individuals diagnosed with ZMC fractures and administered treatment via both two-point and three-point fixation methods. A clinical assessment was performed to evaluate vertical dystopia and enophthalmos, while radiographic analysis was performed to measure zygomatic complex projection and height. The cohort receiving two-point fixation exhibited a statistically significant increase in postoperative vertical dystopia and mean enophthalmos, coupled with a notable reduction in malar projection and height. Conversely, the postoperative outcomes of patients who underwent three-point fixation were superior to those of patients who underwent two-point fixation (Figure 3).

Two-point fixation, typically involving the frontozygomatic suture and either the zygomaticomaxillary buttress or infraorbital rim, offers a less invasive alternative, with shorter operative times and reduced tissue disruption. Gawande et al. [12] found that two-point fixation was sufficient for non-comminuted or minimally displaced fractures, achieving acceptable outcomes. Kim et al. [17] described an altered two-point technique that minimized morbidity while maintaining favorable results. Lee et al. [20] reported satisfactory outcomes in selected cases, emphasizing careful case selection. However, two-point fixation is less effective in controlling rotational displacement, with a higher malar asymmetry in displaced fractures, as noted by Gadkari et al. [12] and Rana et al. [14].

Aesthetic outcomes

Malar symmetry is a critical measure of aesthetic success in ZMC fracture repair. Three-point fixation consistently outperformed two-point fixation in restoring malar contour and projection. Gawande et al. [12] reported malar asymmetry in 13.3% of two-point fixation cases versus 3.3% of three-point fixation cases. Nasr et al. [16] and Gadkari et al. [12] highlighted the superiority of three-point fixation over rotational displacement in minimizing asymmetry. These findings emphasize the advantage of three-point fixation in achieving consistent aesthetic outcomes, particularly in moderate-to-severe fractures.

Complications

Common complications in ZMC fracture management include infraorbital paresthesia, malar flattening, diplopia, and wound infections. Dubron et al. [21] reported infraorbital nerve injury in > 20% of cases, stressing careful surgical handling near the orbital rim. Kim et al. [17] found similar complication rates for two- and three-point fixation in non-comminuted fractures, indicating safety in select cases. However, Nasr et al. [16] noted improved outcomes with three-point fixation in complex fractures, likely because of enhanced stability. Meta-analyses by Jazayeri et al. [13] favored three-point fixation for long-term reliability in severe fractures.

One study indicated that reductions in malar height and vertical dystopia were observed more frequently in situations with a limited number of fixation points [14]. Another analysis contended that three-point fixation exhibited greater stability regarding malar height and mouth aperture than two-point fixation, attributable to the diminished impact of masticatory forces during the recovery period [22]. Conversely, complications manifested less frequently with fewer fixation points. Research has indicated that postoperative facial and neurological complications were less prevalent in patients who underwent two-point fixation as opposed to three-point fixation [15] and that scar formation was more common in two-point fixation than in one-point fixation [23].

Alternative fixation approaches

Single-point fixation, typically at the frontozygomatic suture, is largely obsolete because of inadequate stability and an increased risk of malar asymmetry, as noted by Lee et al. [20]. Four-point fixation, incorporating an additional point such as the zygomatic arch or orbital floor, is reserved for severely comminuted fractures [24]. Choi et al. [24] supported its use in complex injuries, although it increased surgical time and tissue disruption. These approaches highlight the need for tailored strategies based on the fracture characteristics.

Neurological considerations

Infraorbital nerve injuries are a significant concern, with Dubron et al. [21] finding that their incidence was more tied to fracture severity than to the fixation method. However, precise anatomical realignment of the three-point fixation may improve nerve recovery by reducing entrapment near the infraorbital foramen, suggesting an indirect benefit of robust stabilization.

Surgical morbidity and individualized approaches

Minimizing surgical morbidity is the key goal. Elkahwagi et al. [25] advocated minimally invasive single-point fixation in stable fractures to reduce the invasiveness. Cortese et al. [26] proposed the integration of the transconjunctival technique devoid of canthotomy, in conjunction with the transoral maxillary approach and lateral rim skin incision also devoid of canthotomy, for the reduction and stabilization of frontozygomatic fractures that have experienced dislocation, while ensuring no aesthetic compromise in young patients. The literature supports individualized strategies, with three-point fixation preferred for moderate-to-severe fractures and two-point fixation for simpler cases, guided by fracture characteristics and surgeon expertise.

Limitations

This narrative review has a few limitations. First, the inclusion of non-randomized studies (e.g., retrospective cohorts) may introduce selection bias and confounding variables. Second, the heterogeneity in fracture classifications, fixation methods, and outcome measures across studies limits direct comparability. Finally, the review’s narrative (non-systematic) approach lacks quantitative synthesis, potentially overlooking nuanced trends. 

## Conclusions

This narrative review concludes that three-point fixation provides superior stability, malar symmetry, and aesthetic outcomes for complex or displaced ZMC fractures, making it the preferred approach, despite greater surgical invasiveness. Two-point fixation, being less invasive with shorter operative times, proves effective for non-comminuted or minimally displaced fractures but may not adequately control rotational displacement, increasing the risk of malar asymmetry in severe cases. Both techniques show similar complication rates when appropriately applied, although three-point fixation ensures better long-term stability and function in high-energy fractures. Alternative methods, such as single- or four-point fixation, remain limited to specific scenarios owing to inadequate stability or excessive morbidity. The choice of fixation technique depends on fracture complexity, displacement, and patient factors, underscoring the need for individualized treatment plans. Future research should prioritize prospective, multicenter studies to refine minimally invasive approaches that optimize stability while minimizing surgical trauma and enhancing outcomes in ZMC fracture management.
